# Characteristics of exacerbators in the US Bronchiectasis and NTM Research Registry: a cross-sectional study

**DOI:** 10.1183/23120541.00185-2024

**Published:** 2024-11-11

**Authors:** Nicole C. Lapinel, Radmila Choate, Timothy R. Aksamit, Joseph Feliciano, Kevin L. Winthrop, Andreas Schmid, Sebastian Fucile, Mark L. Metersky

**Affiliations:** 1 Northwell Health, New Hyde Park, NY, USA; 2 Louisiana State University Health Sciences Center, New Orleans, LA, USA; 3 University of Kentucky College of Public Health, Lexington, KY, USA; 4 Mayo Clinic, Rochester, MN, USA; 5 Insmed Incorporated, Bridgewater, NJ, USA; 6 Oregon Health and Science University, Portland, OR, USA; 7 University of Kansas Medical Center, Kansas City, KS, USA; 8 University of Connecticut School of Medicine, Farmington, CT, USA

## Abstract

**Background:**

Exacerbations of noncystic fibrosis bronchiectasis (bronchiectasis) are associated with reduced health-related quality of life and increased mortality, likelihood of hospitalisation and lung function decline. This study investigated patient clinical characteristics associated with exacerbation frequency.

**Methods:**

A cross-sectional cohort study of patients ≥18 years with bronchiectasis enrolled in the US Bronchiectasis and Nontuberculous Mycobacteria (NTM) Research Registry (BRR) September 2008–March 2020. Patients were stratified by exacerbation frequency in their 2 years before enrolment. Patient demographics, respiratory symptoms, healthcare resource utilisation, microbiology, modified bronchiectasis severity index (mBSI) and select comorbidities were collected at enrolment. Patient characteristics associated with exacerbation frequency were assessed using a negative binomial model.

**Results:**

The study included 2950 patients (mean age 65.6 years; 79.1% female). Frequency of moderate to severe airway obstruction (forced expiratory volume in 1 s (FEV_1_) % predicted <50%; most recent measure) was 15.9%, 17.8%, and 24.6% in patients with 1, 2, and ≥3 exacerbations *versus* 8.9% in patients with 0 exacerbations; severe disease (mBSI) was 27.8%, 24.2% and 51.1% *versus* 13.2%; respiratory hospitalisation was 24.5%, 33.0% and 36.5% *versus* 4.1%; and *Pseudomonas* *aeruginosa* infection was 18.8%, 23.4% and 35.2% *versus* 11.9%. In multivariable model analysis, respiratory hospitalisation, cough, haemoptysis, *P.*  *aeruginosa*, younger age, lower FEV_1_% predicted, asthma, and gastro-oesophageal reflux disease were associated with more exacerbations.

**Conclusions:**

These findings demonstrate a high disease burden, including increased respiratory symptoms, healthcare resource utilisation, and *P.*  *aeruginosa* infection in patients with bronchiectasis and multiple exacerbations.

## Shareable abstract

**Patients with bronchiectasis and frequent exacerbations have a high disease burden, evidenced by more severe disease and increased respiratory symptoms, airway obstruction, and respiratory hospitalisations.** https://bit.ly/3NW2jmi

## Introduction

Noncystic fibrosis bronchiectasis (bronchiectasis) is a chronic respiratory disease, characterised by cough, sputum production and permanently dilated bronchi and is punctuated by intermittent exacerbations in many patients [1]. Bronchiectasis exacerbations are thought to be triggered by bacterial or viral infections, environmental factors and/or inflammation and present as periodic worsening of disease symptoms [1–4]. Bronchiectasis exacerbations are associated with accelerated lung function decline and increased likelihood of hospitalisation and mortality [5]. Treatment guidelines recommend 14 days of antibiotic therapy for acute exacerbations, with long-term antibiotic treatment recommended for adults with bronchiectasis who have three or more exacerbations per year [5].

The clinical burden associated with bronchiectasis exacerbations has likely been underestimated by studies based on claims data due to the difficulty in capturing data on disease severity [5, 6]. Historically, limited information on the characteristics and course of disease in US adults with bronchiectasis has been available. To address this knowledge gap, the US Bronchiectasis and Nontuberculous Mycobacteria (NTM) Research Registry (BRR) was established as a longitudinal registry of patients with bronchiectasis to improve the understanding of patient characteristics and provide a resource for real-world studies. In the first published description of patients in the registry, 1826 patients enrolled between 2008 and 2014 were analysed in an effort to gain further insight into the characteristics of US patients living with bronchiectasis [7].

The objective of the current study was to utilise data from the BRR to evaluate the association between exacerbations and patient burden by assessing clinical characteristics, healthcare resource utilisation, and disease severity according to bronchiectasis exacerbation frequency during the 2 years prior to enrolment.

## Methods

Data were obtained from the BRR, which is a centralised database of patients with bronchiectasis not associated with cystic fibrosis [7]. At the time of the study (2020), there were 18 clinical sites participating in the BRR throughout the United States. Institutional review board approvals were obtained at each participating site. Patients included were adults aged ≥18 years with bronchiectasis who were enrolled in the BRR between September 2008 and March 2020 and for whom data was available on exacerbations in the 2 years prior to enrolment. Patients with cystic fibrosis were excluded.

Patient characteristics were collected at enrolment and included demographics (age, sex, ethnicity, race, most recent body mass index (BMI), and smoking status), duration of bronchiectasis, number of bronchiectasis exacerbations, respiratory symptoms (fatigue during stable state (when the patient is not experiencing an exacerbation), coughing during stable state, haemoptysis during stable state or exacerbation and wheezing during stable state or exacerbation), hospitalisations in the 2 years prior to enrolment, last recorded forced expiratory volume in 1 s (FEV_1_)% predicted (pre-bronchodilator), any use of bronchial hygiene in the 2 years prior to enrolment, positive tests for *Pseudomonas*  *aeruginosa*, maintenance use of macrolides for bronchiectasis at enrolment, NTM with concurrent treatment at enrolment, and any history of selected comorbidities (COPD, asthma, COPD and asthma, gastro-oesophageal reflux disease (GERD), and otitis and/or rhinosinusitis). Exacerbations were recorded based on the consensus definition for bronchiectasis exacerbations, which is a deterioration in three or more of the following key symptoms for at least 48 h: cough; sputum volume and/or consistency; sputum purulence; breathlessness and/or exercise tolerance; fatigue and/or malaise; haemoptysis; and clinician-determined change in bronchiectasis treatment [8]. Exacerbation data acquired prior to the consensus definition for bronchiectasis exacerbation were based on clinical judgement. Patients were stratified into groups based on bronchiectasis exacerbation frequency (0, 1, 2, and ≥3) in the 2 years prior to enrolment.

Severity of bronchiectasis was assessed using the modified bronchiectasis severity index (mBSI), which was modified from the bronchiectasis severity index (BSI) to align with data collection relative to BRR. Modifications included using data on the number of exacerbations and hospitalisations during the 2 years prior to enrolment (BSI measures the frequency over 1 year) and using presence or absence of dyspnoea when at rest or when active reported by patients at enrolment when Medical Research Council dyspnoea grade was not available. Other inputs to the mBSI calculation (including age, BMI, FEV_1_% predicted, *P.* *aeruginosa* and other organism infection, and radiological severity) could be sourced from data collected at enrolment without requiring modification. An mBSI score of 0 to 4, 5 to 8, and ≥9 was considered mild, moderate, and severe, respectively.

Descriptive statistics were calculated for demographic and clinical characteristics. ANOVA was used to compare values for continuous variables, and chi-square/Mantel-Haenszel chi-square tests were used for categorical variables. An adjusted negative binomial model was used to determine patient characteristics associated with the number of bronchiectasis exacerbations during the 2 years prior to enrolment. Characteristics included in the model were age at enrolment (10-year increments); race; respiratory symptoms, including fatigue, cough, and haemoptysis; hospitalisations for pulmonary illness or exacerbation; FEV_1_% predicted (10% increments); *P.*  *aeruginosa* (≥1 positive culture); use of macrolides; NTM with current treatment; and coexisting conditions, including asthma, COPD, and GERD. For each predictor variable, parameter estimates and incidence rate ratios (IRRs) (± 95% CI) were calculated.

## Results

### Demographic characteristics

After inclusion and exclusion criteria were applied, the study cohort consisted of 2950 patients (supplementary figure 1). Mean age was 65.6 years, 79.1% of the cohort was female, and the mean duration of bronchiectasis diagnosis was 7.6 years (table 1). In the 2 years prior to enrolment, 41.2% of patients (n=1214) had 0 bronchiectasis exacerbations; 23.4% (n=691) had 1 exacerbation; 14.8% (n=438) had 2 exacerbations; and 20.6% (n=607) had 3 or more exacerbations.

**FIGURE 1:**
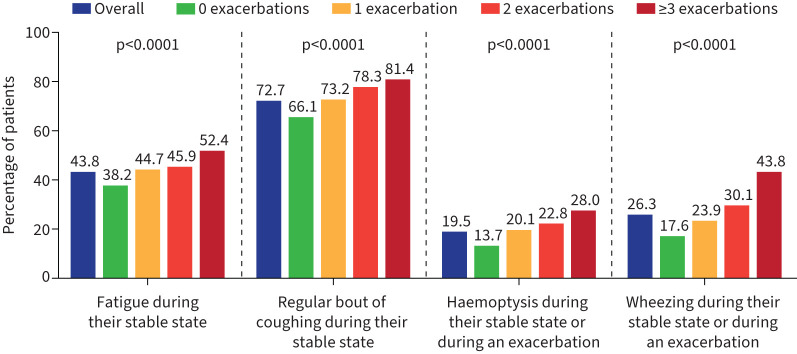
Respiratory symptoms in the overall cohort and stratified by number of exacerbations. p*-*values shown are for comparison across four exacerbation groups.

**TABLE 1:** Demographic characteristics in the overall cohort and stratified by number of exacerbations

	Data available (n)	Overall N=2950	0 exacerbations n=1214 (41.2%)	1 exacerbation n=691 (23.4%)	2 exacerbations n=438 (14.8%)	≥3 exacerbations n=607 (20.6%)	p*-*value^#^
**Age, years**	2924	65.6 (13.4)	67.3 (12.3)	65.1 (13.7)	65.6 (14.2)	63.1 (14.0)	<0.0001
**Age at bronchiectasis diagnosis, years**	2203	58.4 (16.6)	60.9 (15.6)	59.3 (16.1)	58.1 (17.0)	53.3 (17.2)	<0.0001
**Bronchiectasis diagnosis duration, years**	2165	7.6 (13.4)	6.7 (10.1)	6.7 (9.7)	7.6 (9.8)	10.0 (12.1)	<0.0001
**Sex, n (%)** Female Male	2943	2328 (79.1) 615 (20.9)	951 (78.5) 260 (21.5)	550 (79.7) 140 (20.3)	349 (79.9) 88 (20.1)	478 (79.0) 127 (21.0)	0.7233
**Race, n (%)** White Black/African American Asian Other^¶^ Unknown	2950	2659 (90.1) 74 (2.5) 101 (3.4) 74 (2.5) 42 (1.4)	1084 (89.3) 30 (2.5) 56 (4.6) 25 (2.1) 19 (1.6)	623 (90.2) 17 (2.5) 23 (3.3) 17 (2.5) 11 (1.6)	408 (93.2) 7 (1.6) 12 (2.7) 7 (1.6) 4 (0.9)	544 (89.6) 20 (3.3) 10 (1.6) 25 (4.1) 8 (1.3)	0.0054
**Hispanic ethnicity, n (%)** No Yes Unknown	2950	2456 (83.3) 121 (4.1) 373 (12.6)	962 (79.2) 40 (3.3) 212 (17.5)	584 (84.5) 26 (3.8) 81 (11.7)	378 (86.3) 19 (4.3) 41 (9.4)	532 (87.6) 36 (5.9) 39 (6.4)	0.1874
**Smoking status, n (%)** Ever smoker Never smoker Unknown	2950	1227 (41.6) 1698 (57.6) 25 (0.8)	513 (42.3) 687 (56.6) 14 (1.2)	293 (42.4) 389 (56.3) 9 (1.3)	179 (40.9) 258 (58.9) 1 (0.2)	242 (39.9) 364 (60.0) 1 (0.2)	0.6173
**BMI, kg·m^−2^**	2489	22.9 (4.9)	22.5 (4.6)	22.9 (4.7)	23.3 (5.1)	23.5 (5.5)	0.0007

Data are presented as mean (sd) unless otherwise stated. Unknown or missing data were not included in any comparisons. BMI: body mass index. ^#^: p*-*values shown are for comparison across four exacerbation groups. ^¶^: “Other” includes Native Hawaiian or other Pacific Islander, American Indian or Alaska Native, or other.

### Respiratory symptoms, spirometry, and disease severity

Patients with 1, 2, and ≥3 bronchiectasis exacerbations in the 2 years prior to enrolment had a higher frequency of respiratory symptoms including fatigue, regular bout of coughing, haemoptysis, and wheezing compared with those who had 0 exacerbations (figure 1). Symptoms were also more common in patients with a greater number of exacerbations.

**FIGURE 2:**
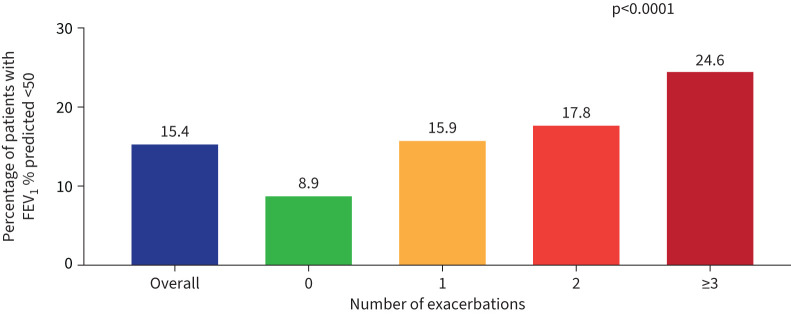
Forced expiratory volume in 1 s (FEV_1_)% predicted <50% in the overall cohort and stratified by number of exacerbations. p*-*value is for comparison across four exacerbation groups.

Patients with bronchiectasis exacerbations in the 2 years prior to enrolment had a higher frequency of moderate to severe airway obstruction (FEV_1_% predicted <50%) compared with patients who had 0 exacerbations (figure 2). The frequency of moderate to severe airway obstruction increased with the number of bronchiectasis exacerbations in the 2 years prior to enrolment.

**FIGURE 3:**
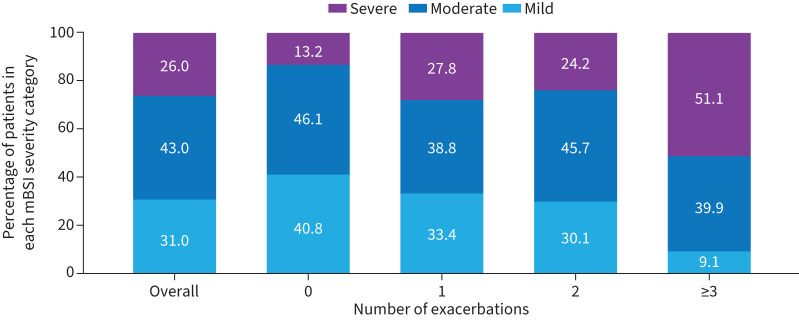
Modified bronchiectasis severity index (mBSI) in the overall cohort and stratified by number of exacerbations. BSI was modified by using data on the number of exacerbations in the 2 years prior to enrolment; where dyspnoea grade was not available, variables related to the patient experiencing dyspnoea at rest or when active were used. mBSI was calculated using all available data on any of the nine parameters and summing values for available parameters only. An mBSI score of 0–4 was mild, 5–8 was moderate and ≥9 was severe.

Mean (sd) mBSI was higher in the groups with a greater number of bronchiectasis exacerbations in the 2 years prior to enrolment: 6.6 (3.6), 5.4 (2.7), 6.5 (3.6), 6.6 (3.8) and 9.0 (3.7) for the overall, 0, 1, 2, and ≥3 bronchiectasis exacerbations groups, respectively. Patients with ≥3 bronchiectasis exacerbations in the 2 years prior to enrolment had the highest frequency of severe disease based on the mBSI, compared with patients who had fewer exacerbations (figure 3).

### Healthcare resource utilisation

**FIGURE 4:**
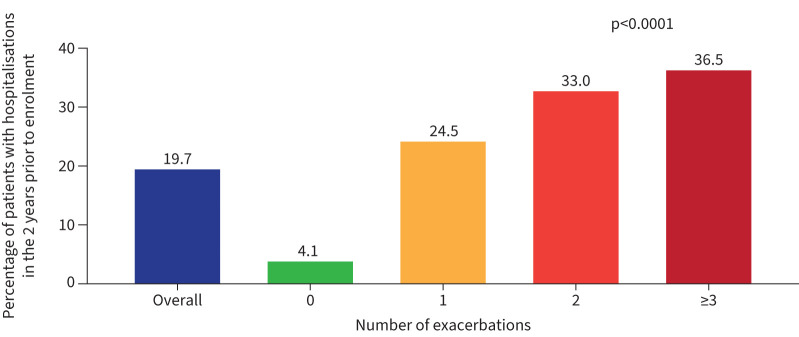
Proportion of patients with hospitalisations in the overall cohort and stratified by number of exacerbations. p*-*value shown is for comparison across four exacerbation groups. Hospitalisations are for pulmonary illness or exacerbation.

A greater proportion of patients reporting bronchiectasis exacerbations in the 2 years prior to enrolment were hospitalised (figure 4; 24.5% for 1 exacerbation, 33.0% for 2 exacerbations, and 36.5% for ≥3 exacerbations). Patients reporting exacerbations in the 2 years prior to enrolment had a larger mean number of hospitalisations compared with patients who had 0 bronchiectasis exacerbations (table 2).

**TABLE 2:** Healthcare resource utilisation and microbiology in the overall cohort and stratified by number of exacerbations

	Data available (n)	Overall N=2950	0 exacerbations n=1214 (41.2%)	1 exacerbation n=691 (23.4%)	2 exacerbations n=438 (14.8%)	≥3 exacerbations n=607 (20.6%)	p*-*value^#^
**Healthcare resource utilisation**							
Number of hospitalisations, mean (sd)	2915	0.30 (0.73)	0.06 (0.34)	0.27 (0.51)	0.49 (0.83)	0.69 (1.14)	<0.0001
Use of bronchial hygiene,^¶^ n (%) Yes No	2822	1623 (57.5) 1199 (42.5)	572 (49.0) 596 (51.0)	405 (62.1) 247 (37.9)	257 (62.2) 156 (37.8)	389 (66.0) 200 (34.0)	<0.0001
**Microbiology**							
One or more cultures positive for *Pseudomonas aeruginosa,* n (%) Yes No	2926	586 (20.0) 2340 (80.0)	144 (11.9) 1063 (88.1)	129 (18.8) 556 (81.2)	101 (23.4) 331 (76.6)	212 (35.2) 390 (64.8)	<0.0001
Maintenance use of macrolides, n (%) Yes No	2938	528 (18.0) 2410 (82.0)	200 (16.5) 1012 (83.5)	103 (15.0) 585 (85.0)	88 (20.2) 347 (79.8)	137 (22.7) 466 (77.3)	0.0008
NTM with current treatment,^+^ n (%) Yes No	2669	616 (23.1) 2053 (76.9)	302 (27.8) 786 (72.2)	160 (25.4) 469 (74.6)	70 (17.9) 322 (82.1)	84 (15.0) 476 (85.0)	<0.0001

Data are presented as mean (sd) or n (%). Unknown or missing data were not included in any comparisons. NTM: nontuberculous mycobacteria. ^#^: p*-*values shown are for comparison across four exacerbation groups. ^¶^: modalities included in the data-collection form were positive expiratory pressure, chest percussion/postural drainage, directed cough/active cycle of breathing, high-frequency chest wall oscillation, other measures, and unknown. ^+^: defined as NTM (+) using one or more positive NTM cultures and/or history of NTM and NTM treatment during 2 years prior to enrolment.

### Microbiology

Patients with bronchiectasis exacerbations in the 2 years prior to enrolment had a greater proportion of positive cultures for *P.*  *aeruginosa* compared with patients with 0 exacerbations (table 2). The proportion of patients with a positive culture for *P.*  *aeruginosa* increased with the number of bronchiectasis exacerbations (table 2; 18.8% for 1 exacerbation, 23.4% for 2 exacerbations, and 35.2% for ≥3 exacerbations). Maintenance use of macrolides at enrolment was more common in patients with 2 and ≥3 bronchiectasis exacerbations in the 2 years prior to enrolment, compared with patients with 0 and 1 bronchiectasis exacerbations (table 2; 20.2% and 22.7% *versus* 16.5% and 15.0%). Patients with 2 and ≥3 bronchiectasis exacerbations in the 2 years prior to enrolment had the lowest proportion undergoing treatment for NTM, compared with those with 0 and 1 exacerbations (table 2, 17.9% and 15.0% *versus* 27.8% and 25.4%).

### Comorbidities

**FIGURE 5:**
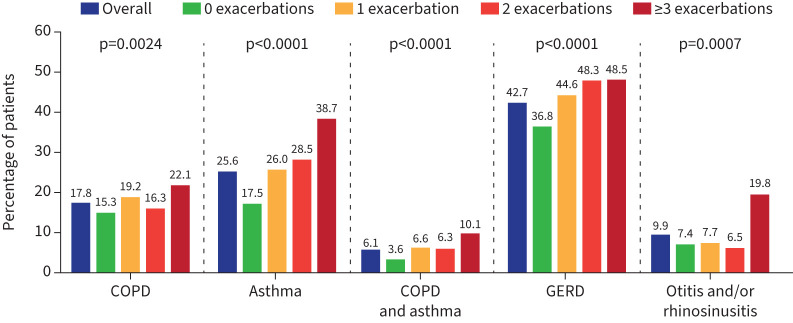
Comorbidities in the overall cohort and stratified by number of exacerbations. p*-*values shown are for comparison across four groups. GERD: gastro-oesophageal reflux disease.

GERD, asthma, COPD, otitis and/or rhinosinusitis, and COPD and asthma were common comorbidities in this patient cohort (figure 5). These comorbidities were present in a greater proportion of patients who had more frequent bronchiectasis exacerbations. For example, a greater proportion of patients with 1 exacerbation in the 2 years prior to enrolment had asthma compared with patients with 0 exacerbations (26.0% *versus* 17.5%) and the proportion of patients with asthma increased with the number of exacerbations (26.0% for 1 exacerbation, 28.5% for 2 exacerbations, and 38.7% for ≥3 exacerbations). The proportion of patients with otitis and/or rhinosinusitis was similar in patients with 0, 1, or 2 bronchiectasis exacerbations (7.4%, 7.7%, and 6.5%, respectively) but was higher for patients with ≥3 exacerbations (19.8%).

### Multivariable model analysis

**FIGURE 6:**
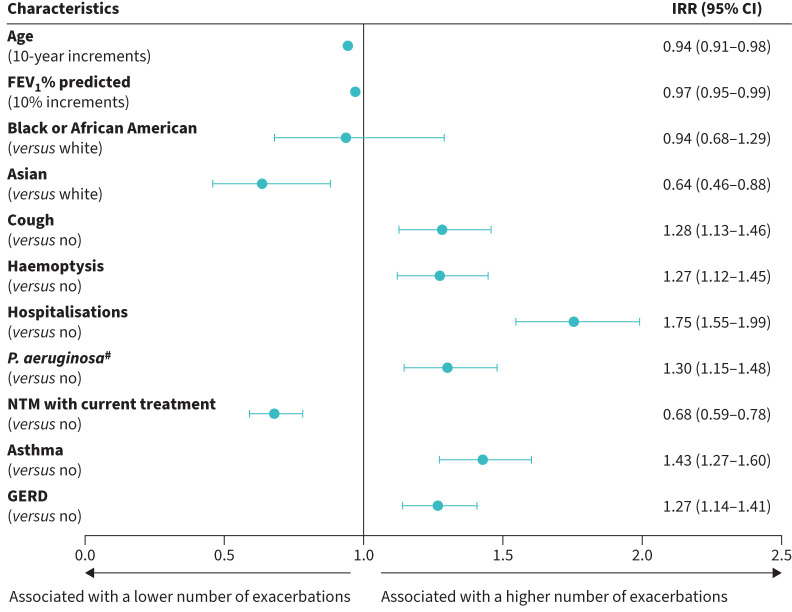
Adjusted negative binomial model for number of bronchiectasis exacerbations. FEV_1_: forced expiratory volume in 1 s; GERD: gastro-oesophageal reflux disease; IRR: incidence rate ratio; NTM: nontuberculous mycobacteria; *P.*  *aeruginosa*: *Pseudomonas* *aeruginosa.* ^#^: ≥1 positive culture.

The adjusted negative binomial model identified cough, haemoptysis, hospitalisation, positive culture for *P.*  *aeruginosa*, asthma, and GERD as being associated with a higher number of bronchiectasis exacerbations in the 2 years prior to enrolment (figure 6). Asian race and NTM infection with current treatment were associated with a lower number of exacerbations. Controlling for other variables in the model, age was found to be negatively associated with the number of exacerbations with an IRR of 0.94 (95% CI 0.91–0.98) for every 10-year increase in age (a 5.7% decrease in IRR for every 10-year increase in age). FEV_1_% predicted was found to be negatively associated with the number of exacerbations with an IRR of 0.97 (95% CI 0.95–0.99) for every 10% increase in FEV_1_% predicted (that is a 3.0% decrease in IRR for exacerbations for every 10% increase in FEV_1_% predicted).

## Discussion

The results of this real-world patient registry study demonstrate the high disease burden and increased healthcare resource utilisation in patients with bronchiectasis and frequent exacerbations and provides insight into patient characteristics associated with frequent exacerbations. More than half of the patients in this study had at least 1 bronchiectasis exacerbation in the 2 years prior to enrolment, and one-fifth had ≥3 exacerbations. These proportions are similar to those reported in past studies. In a multicentre European study of 608 patients with bronchiectasis, the annual exacerbation frequency was 1.8 per patient per year [9], and in a larger European cohort of 2572 patients approximately 70% of patients had experienced one or more bronchiectasis exacerbations per year [10].

The frequency of respiratory symptoms and moderate to severe airway obstruction was higher for patients with bronchiectasis exacerbations during the 2 years prior to enrolment than for those without. This finding is consistent with previous studies which reported higher exacerbation frequency associated with increased inflammation and progressive lung damage. Specifically, more frequent exacerbations are associated with poor health-related quality of life, lung function decline, mortality, and greater likelihood of hospitalisation [9, 10].

Disease severity can be defined according to BSI, which was developed to predict future mortality risk. It is, however, important to consider that the reality of disease for patients with mild, moderate, or severe BSI is likely to be more complex. For example, a patient who is experiencing frequent exacerbations but has a lower BSI score would not be considered as having “mild disease” in clinical practice. Patient characteristics that are included in the BSI are severe dyspnoea, FEV_1_ <30% predicted, prior hospital admissions, colonisation with other pathogenic organisms, *P.*  *aeruginosa* colonisation, and bronchiectasis involving three or more lobes [9]. In the current study, patients with ≥3 bronchiectasis exacerbations were nearly four times more likely to have severe disease (as assessed by mBSI) compared with patients with 0 exacerbations. However, exacerbations are also components of the mBSI and are therefore mathematically coupled to the mBSI severity score for each patient. As such, it is not surprising that the mBSI score is higher for patients with more frequent exacerbations. In a previous study from the BRR investigating the association between *P.*  *aeruginosa* infection and disease severity according to mBSI, which are similarly mathematically coupled, when *P.*  *aeruginosa* infection was excluded from the mBSI calculation, the association between infection and more severe mBSI score was maintained, although the effect size was smaller [11]. Nevertheless, the current study demonstrates the association between exacerbations and other measures of severity, such as respiratory symptoms, comorbidities, and hospitalisations and highlights the importance of studying exacerbations as a measure of disease severity.

With respect to healthcare resource utilisation, both the mean number of respiratory hospitalisations and the proportion of patients with any respiratory hospitalisation was higher in patients with more exacerbations. Patients with ≥3 exacerbations in the 2 years prior to enrolment had more than 10 times the mean number of hospitalisations of patients with 0 exacerbations. This association between more frequent exacerbations and hospitalisations has previously been reported; in a European cohort study of 2572 patients, no patients without exacerbations had been hospitalised, whereas 57% of patients with ≥3 exacerbations per year had been hospitalised [10].

A greater proportion of patients with ≥3 bronchiectasis exacerbations in the 2 years prior to enrolment had positive cultures for *P.*  *aeruginosa* and maintenance macrolide use. *P.*  *aeruginosa* chronic infection has previously been linked to exacerbation risk and is associated with increased mortality in patients with frequent exacerbations (hazard ratio 2.03; 95% CI 1.36–3.03) and hospitalisations (OR 2.28; 95% CI 1.69–3.08) [12]. However, longitudinal studies of the natural history of bronchiectasis and *P.* *aeruginosa* infection would be required in order to establish a causal relationship. NTM with concurrent treatment was present in a smaller proportion of patients with 2 and ≥3 exacerbations in the 2 years prior to enrolment, compared with patients with 0 and 1 exacerbations. This was an interesting finding, as previous studies have reported conflicting results as they relate to NTM pulmonary disease in association with more frequent exacerbations [13–15]. The difference may be partly related to previous studies investigating NTM in association with other pathogens and differences in geography, ethnicity, and practice patterns. It is also important to consider that the current study used registry data obtained from specialist NTM/bronchiectasis centres and the treatment practices at these centres could affect outcomes related to exacerbation frequency. In the European cohort study of 2572 patients, which most closely resembles the cohort in the current study, NTM infection was not included in the analysis of factors associated with exacerbation frequency [10].

The multivariable analysis performed in the current study identified that cough, haemoptysis, hospitalisation, *P.*  *aeruginosa*, asthma, and GERD were all associated with a higher number of bronchiectasis exacerbations in the 2 years prior to enrolment, and FEV_1_% predicted and age were negatively associated with exacerbation frequency. A previous study to identify independent risk factors for future exacerbations similarly found that FEV_1_% predicted was negatively associated with exacerbations in follow-up; however, age and asthma were not [10]. In that previous study, although there was a similar trend towards older patients having fewer exacerbations and patients with asthma having more exacerbations, neither were significantly associated with exacerbations in their multivariable analysis [10]. For asthma, the lack of significant association with exacerbations may have been due to a lack of statistical power, with only 9% of patients (n=226) in that study having asthma, compared with 26% of patients in this study. Although the present study did not assess mortality, more frequent exacerbations have previously been linked with a greater mortality risk; in the European cohort study of 2572 patients, the hazard ratio for mortality was nearly double for patients with ≥3 *versus* 0 exacerbations per year [10]. Patient registries such as the BRR are important for further defining the link between exacerbations and mortality for patients with bronchiectasis.

In the current study, patients with one bronchiectasis exacerbation in the 2 years prior to enrolment had higher proportions of COPD, asthma, COPD and asthma, and GERD than patients with no exacerbations. For patients with three or more bronchiectasis exacerbations, the frequency of otitis and/or rhinosinusitis was more than 2.5 times higher than in patients with no exacerbations. Multimorbidity in patients with bronchiectasis has previously been identified, with the most common comorbidities in a previous report including GERD (34.3%), hypertension (27.5%), hyperlipidaemia (20.1%), and COPD (17.1%) [16]; proportions of patients with GERD and COPD were similar in the current study. Other reported comorbidities include cardiovascular diseases, psychological disorders and pulmonary hypertension [16], although these comorbidities are not included in the BRR and could not be assessed in the current study.

Despite the significant morbidity associated with bronchiectasis in the US, there are limited data available on the characteristics of patients with bronchiectasis. The findings of this study provide new insight into the clinical and demographic characteristics of patients with bronchiectasis and will facilitate future research building on these findings, including longitudinal studies of the BRR, with the ultimate aim of improving patient outcomes.

### Limitations

As a cross-sectional study, the timing of exacerbations and characteristics could not be assessed, and longitudinal studies are required in order to address this. These results were collected from tertiary referral centres with an interest in bronchiectasis enrolled in the registry and as such may not be fully representative of the bronchiectasis patient population throughout the United States. Additionally, these results cannot be extrapolated to cohorts of patients with bronchiectasis residing outside the US without further study. Given the retrospective nature of data collection at enrolment, the data may be subject to reporting bias, due to incorrect recall, as well as errors in medical recordkeeping, and medical-chart abstraction. The mBSI score used to assess disease severity was modified from BSI according to the structure of data collection for the BRR.

### Conclusions

These findings demonstrate a high disease burden in patients with bronchiectasis and frequent exacerbations, including increased respiratory symptoms, severity of disease according to mBSI, respiratory hospitalisations, *P.* *aeruginosa* infection, and moderate to severe airway obstruction. This study identified important characteristics of patients with more frequent exacerbations, which may help define cohorts for prospective studies on the impact of exacerbations. In clinical practice, these results provide insight into the broader characteristics of patients with bronchiectasis by exacerbation frequency in a large population.

## Supplementary material

10.1183/23120541.00185-2024.Supp1 **Please note:** supplementary material is not edited by the Editorial Office, and is uploaded as it has been supplied by the author.
Supplementary material 00185-2024.SUPPLEMENT

## References

[1] Amati F, Simonetta E, Gramegna A, et al. The biology of pulmonary exacerbations in bronchiectasis. Eur Respir Rev 2019; 28: 190055. doi:10.1183/16000617.0055-2019 31748420 PMC9488527

[2] Flume PA, Chalmers JD, Olivier KN. Advances in bronchiectasis: endotyping, genetics, microbiome, and disease heterogeneity. Lancet 2018; 392: 880–890. doi:10.1016/S0140-6736(18)31767-7 30215383 PMC6173801

[3] Gao Y-H, Guan W-J, Xu G, et al. The role of viral infection in pulmonary exacerbations of bronchiectasis in adults: a prospective study. Chest 2015; 147: 1635–1643. doi:10.1378/chest.14-1961 25412225 PMC7094490

[4] Choi H, Chalmers JD. Bronchiectasis exacerbation: a narrative review of causes, risk factors, management and prevention. Ann Transl Med 2023; 11: 25. doi:10.21037/atm-22-3437 36760239 PMC9906191

[5] Polverino E, Goeminne PC, McDonnell MJ, et al. European Respiratory Society guidelines for the management of adult bronchiectasis. Eur Respir J 2017; 50: 1700629. doi:10.1183/13993003.00629-2017 28889110

[6] Joish VN, Spilsbury-Cantalupo M, Operschall E, et al. Economic burden of non-cystic fibrosis bronchiectasis in the first year after diagnosis from a US health plan perspective. Appl Health Econ Health Policy 2013; 11: 299–304. doi:10.1007/s40258-013-0027-z 23580074

[7] Aksamit TR, O'Donnell AE, Barker A, et al. Adult patients with bronchiectasis: a first look at the US bronchiectasis research registry. Chest 2017; 151: 982–992. doi:10.1016/j.chest.2016.10.055 27889361 PMC6026266

[8] Hill AT, Haworth CS, Aliberti S, et al. Pulmonary exacerbation in adults with bronchiectasis: a consensus definition for clinical research. Eur Respir J 2017; 49: 1700051. doi:10.1183/13993003.00051-2017 28596426

[9] Chalmers JD, Goeminne P, Aliberti S, et al. The bronchiectasis severity index. An international derivation and validation study. Am J Respir Crit Care Med 2014; 189: 576–585. doi:10.1164/rccm.201309-1575OC 24328736 PMC3977711

[10] Chalmers JD, Aliberti S, Filonenko A, et al. Characterization of the “frequent exacerbator phenotype” in bronchiectasis. Am J Respir Crit Care Med 2018; 197: 1410–1420. doi:10.1164/rccm.201711-2202OC 29357265

[11] Choate R, Aksamit TR, Mannino D, et al. *Pseudomonas aeruginosa* associated with severity of non-cystic fibrosis bronchiectasis measured by the modified bronchiectasis severity score (BSI) and the FACED: the US bronchiectasis and NTM Research Registry (BRR) study. Respir Med 2021; 177: 106285. doi:10.1016/j.rmed.2020.106285 33401148

[12] Araújo D, Shteinberg M, Aliberti S, et al. The independent contribution of *Pseudomonas aeruginosa* infection to long-term clinical outcomes in bronchiectasis. Eur Respir J 2018; 51: 1701953. doi:10.1183/13993003.01953-2017 29386336

[13] Yin H, Gu X, Wang Y, et al. Clinical characteristics of patients with bronchiectasis with nontuberculous mycobacterial disease in mainland China: a single center cross-sectional study. BMC Infect Dis 2021; 21: 1216. doi:10.1186/s12879-021-06917-8 34872515 PMC8650543

[14] Lin CY, Huang HY, Hsieh MH, et al. Impacts of nontuberculous mycobacteria isolates in non-cystic fibrosis bronchiectasis: a 16-year cohort study in Taiwan. Front Microbiol 2022; 13: 868435. doi:10.3389/fmicb.2022.868435 35509319 PMC9058169

[15] Kwak N, Lee JH, Kim HJ, et al. New-onset nontuberculous mycobacterial pulmonary disease in bronchiectasis: tracking the clinical and radiographic changes. BMC Pulm Med 2020; 20: 293. doi:10.1186/s12890-020-01331-3 33172424 PMC7653824

[16] McDonnell MJ, Aliberti S, Goeminne PC, et al. Comorbidities and the risk of mortality in patients with bronchiectasis: an international multicentre cohort study. Lancet Respir Med 2016; 4: 969–979. doi:10.1016/S2213-2600(16)30320-4 27864036 PMC5369638

