# Supplementary material for: Characteristics of exacerbators in the US Bronchiectasis and NTM Research Registry: a cross-sectional study
Source: ERJ Open Res. 2024 Nov 11;10(6):00185-2024. doi: 10.1183/23120541.00185-2024 (PMC11551853; doi:10.1183/23120541.00185-2024)
Supplement: Supplementary file 1 [file 00185-2024.SUPPLEMENT.pdf]

Supplemental tables and figures

Supplemental Figure 1. Cohort attrition

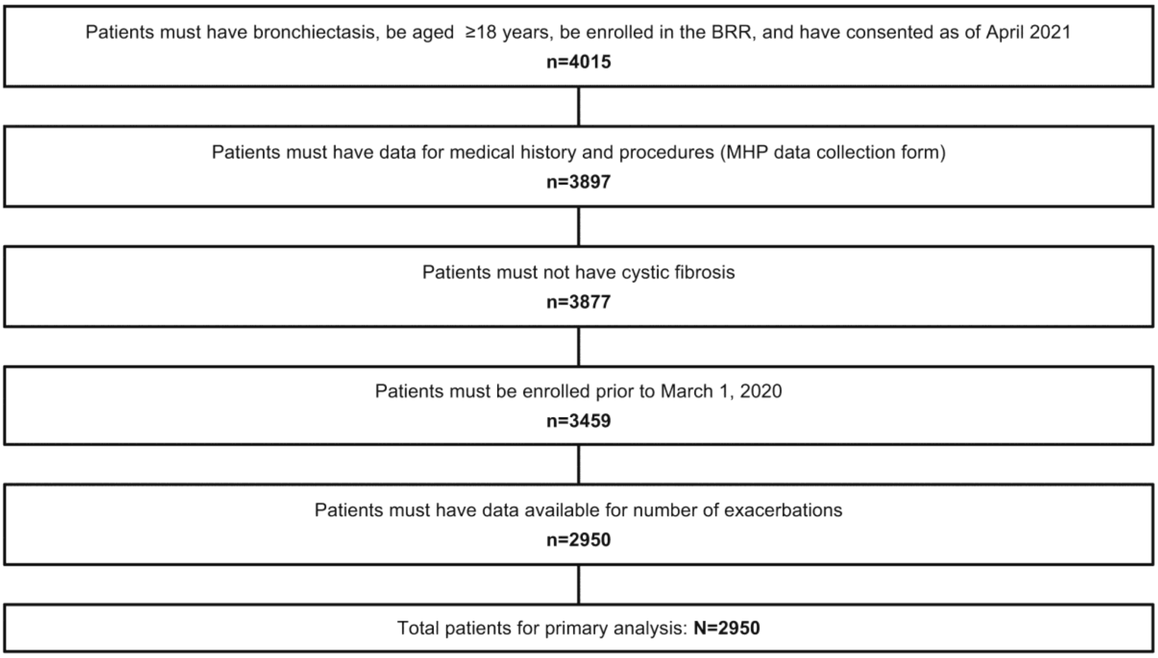

BRR, US Bronchiectasis Research Registry; MHP, medical history and procedures.
